# Prenatal alcohol exposure and child sleep problems: A family‐based quasi‐experimental study

**DOI:** 10.1002/jcv2.12111

**Published:** 2022-11-02

**Authors:** Ingunn Olea Lund, Eivind Ystrom

**Affiliations:** ^1^ Department of Mental Disorders Norwegian Institute of Public Health Oslo Norway; ^2^ Department of Psychology Faculty of Social Sciences University of Oslo Oslo Norway; ^3^ PROMENTA Research Center Department of Psychology Faculty of Social Sciences University of Oslo Oslo Norway; ^4^ PharmacoEpidemiology and Drug Safety Research Group, School of Pharmacy, & PharmaTox Strategic Initiative, Faculty of Mathematics and Natural Sciences University of Oslo Oslo Norway

**Keywords:** alcohol, instrumental variable, medical birth registry of Norway, prenatal exposure, sibling design, sleep, the Norwegian mother, father and child cohort study

## Abstract

**Background:**

We examine whether associations between prenatal exposure to hazardous maternal alcohol consumption during the first trimester of pregnancy and sleep problems in young children represent a causal association.

**Methods:**

The population‐based sample consists of 15,911 mothers with 30,395 offspring from the Norwegian Mother, Father, and Child Cohort Study (MoBa) and the Medical Birth Registry of Norway (MBRN). Women self‐reported pre‐pregnancy alcohol consumption and consumption during the first trimester of pregnancy twice: at gestational weeks 17 and 30. Mothers reported their children's sleep problems, when they were 1.5 and 3 years (mean = 50; SD = 10). We tested models adjusting for (1) measured confounders, (2) unmeasured familial risk factors by sibling design, and (3) maternal hazardous drinking in the 3 months prior to pregnancy as an instrumental variable within the sibling design.

**Results:**

Children of mothers with hazardous drinking during the first trimester were at increased risk of sleep problems at 1.5 (*β* = 1.14, 95%CI 0.04–2.25) and 3 (*β* = 2.86, 95%CI 1.85–3.87) years of age. These associations were reduced to close to zero and non‐significant at 1.5 (*β* = −0.32, 95%CI −1.91–1.26) and 3 (*β* = 0.06, 95%CI −1.56–1.64) years when controlling for both familial and measured environmental risk factors.

**Conclusions:**

There is a moderate association between maternal hazardous drinking during pregnancy and offspring sleep problems up to age three. This association is explained by risk factors differing between families and does not reflect a cause‐effect relationship.

## Graphical abstract

We examined whether associations between prenatal exposure to hazardous maternal alcohol consumption during the first trimester of pregnancy and sleep problems in young children represent a causal association. Using a population‐based sample of 15,911 mothers with 30,395 offspring, we found that the association between maternal hazardous drinking during pregnancy and offspring sleep problems up to age three does not reflect a cause‐effect relationship. The findings help to advance the understanding of associations between maternal hazardous drinking during pregnancy and child sleep problems and inform future intervention and prevention research.

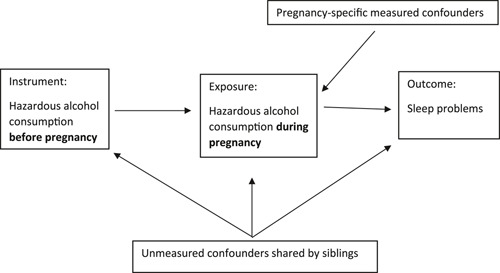

**Key points**

- Maternal drinking during pregnancy is associated with child sleep problems, but the collective research findings show conflicting results. It remains challenging to disentangle the true association, information crucial to inform intervention and prevention research.
- Using a population‐based sample of 15,911 mothers with 30,395 offspring we found that the association between maternal hazardous drinking during pregnancy and offspring sleep problems up to age 3 does not reflect a cause‐effect relationship.
- The findings help to advance the understanding of associations between maternal hazardous drinking during pregnancy and child sleep problems and inform future intervention and prevention research.

**Abbreviations**
Audit‐C: the alcohol use disorder identification test consumption
CBCL: child behavior checklist for preschool children
CI: Confidence Interval
FAS: fetal alcohol syndrome
FASD: fetal alcohol spectrum disorders
GRM‐IRT: graded response item response theory model
MoBa: the Norwegian mother, father, and child cohort study

## INTRODUCTION

About 10% of young children have sleep problems (Byars et al., 2012). Sleep is imperative to function, and particularly important during the first years of life where insufficient and lack of high‐quality sleep may compromise brain development (Lokhandwala & Spencer, 2022). Further, it may negatively impact cognitive, emotional, behavior and physical outcomes during childhood and into adulthood (Fatima et al., 2015; Jiang, 2019; Reynaud et al., 2018; Sivertsen et al., 2015; Spruyt, 2019); the magnitude of sleep problems in young children (Byars et al., 2012) and the severe outcomes associated with it (Ednick et al., 2009; Fatima et al., 2015; Jiang, 2019; Reynaud et al., 2018; Sivertsen et al., 2015; Spruyt, 2019) underscores the need for reliable knowledge about risk factors associated with child sleep problems that can inform intervention and prevention research.

Maternal drinking during pregnancy is a risk factor associated with offspring sleep problems (Chandler‐Mather et al., 2021). However, the collective research findings remain ambiguous, with some studies suggesting there is an association (Alvik et al., 2011; Chandler‐Mather et al., 2021; Harskamp‐van Ginkel et al., 2020; Pesonen et al., 2009; Shang et al., 2006; Troese et al., 2008), while others do not (Morales‐Muñoz et al., 2018; Stone et al., 2010). To date, most studies have limiting factors in their research designs, such as evaluating the effect of prenatal exposure to alcohol on child sleep problems using small samples (Troese et al., 2008). Further, most study designs fail to disentangle the effect of prenatal exposure from other factors that may also influence offspring sleep outcomes (Alvik et al., 2011; Chandler‐Mather et al., 2021; Harskamp‐van Ginkel et al., 2020; Morales‐Muñoz et al., 2018; Pesonen et al., 2009; Shang et al., 2006; Stone et al., 2010; Troese et al., 2008). This may lead to erroneous conclusions, and consequently, that intervention research strategies are being based on erroneous results, a major waste of research resources (Ioannidis et al., 2014).

In our study, we use an established alternative method to provide more informative answers about the role of prenatal exposure to alcohol on young children's sleep problems by utilizing family‐based quasi‐experimental research designs (D'onofrio et al., 2013; Lahey & D’Onofrio, 2010). This approach control for unmeasured familial (i.e., genetic and shared environmental) factors shared within sibling pairs. This is important given that it is well established that genetic and shared environmental factors influence both alcohol use and psychiatric disorders (Kendler et al., 2003; Ystrom et al., 2022), including sleep problems (Moore et al., 2011). To resolve the controversies in the literature and offer more reliable information to inform intervention research, we use two approaches to control for unmeasured confounding: first, we compare siblings raised in the same family but exposed to different levels of maternal drinking, this excludes confounding of the exposure with other environmental factors shared by the siblings and control for genetic confounding (D'onofrio et al., 2013). Second, we use instrumental variables to control for confounding (Davies et al., 2017); the assumption is that if hazardous drinking before pregnancy is associated with the outcome, this will be due to other factors than a direct intrauterine effect (Lund et al., 2019). We estimate the effect of hazardous drinking during pregnancy on offspring sleep outcomes when children are 1.5 and 3 years old, considering both environmental and genetic risks. Hazardous alcohol consumption during the first trimester of pregnancy is particularly harmful to the developing fetus (Nykjaer et al., 2014), this drinking pattern and time period is therefore the focus of the present study.

## METHODS

### Sample, data sources, and ethics

We use data from the Norwegian Mother, Father, and Child Cohort Study (MoBa), a prospective, ongoing pregnancy cohort study described in detail elsewhere (Magnus et al., 2016). Participants were recruited from 1999 to 2008 at a routine ultrasound examination offered to all pregnant women in Norway during gestational week 17–18, 41% of eligible women participated. The total sample includes more than 114,000 children, 95,000 mothers, and 75,000 fathers. The current study encompassed 15,911 mothers with 30,395 offspring full siblings. We used data collected during the 17th and the 30th week of gestation and when children were 1.5 and 3 years old. We also obtained information from the Medical Birth Registry of Norway (Irgens, 2000). We used version 9 of the quality‐assured MoBa data files, released in 2015. All study participants provided written informed consent. MoBa has a license from the Norwegian Data Inspectorate, and this study was approved by the Regional Committee for Medical Research Ethics.

## MEASURES

### Hazardous alcohol consumption

The Alcohol Use Disorder Identification Test Consumption (AUDIT‐C; Bush et al., 1998) was used to index hazardous alcohol consumption, defined as the quantity and/or pattern of alcohol consumption that places drinkers at risk for negative health outcomes (Reid et al., 1999; Saunders et al., 1993). The scale consists of three items summed to a score between 0 and 12: Frequency of drinking was measured using a scale ranging from “never” to “approximately 6–7 times per week”; the usual amount of drinking was measured using a scale ranging from “less than 1” to “10 or more”; and frequency of binge drinking (5 or more units of alcohol per drinking occasion), was measured using a scale ranging from “never” to “several times per week”. In MoBa, one unit of alcohol is defined as “1.5 cl (12.8 g) of pure alcohol” (Knudsen et al., 2015). In the questionnaires administered during pregnancy weeks 17 and 30, women reported alcohol consumption in the three months before pregnancy and during the first trimester. We used the average of these two reports for pre‐pregnancy and first trimester alcohol use, respectively. For women, a cut‐off of three on AUDIT‐C may be used to indicate the risk of alcohol‐related problems (Bradley et al., 2003) This cut‐off has high sensitivity and specificity for identifying alcohol‐related problems also in pregnant populations (Dawson et al., 2005).

### Child sleep problems

We used two items from the Child Behavior Checklist version for pre‐school children (CBCL/1.5–5) to assess child sleep problems at ages 1.5 and 3 years (29). CBCL/1.5–5 consists of 99 items that describe child behavior in the preceding 2 months, including a sleep problems subscale. The items used were: “Doesn't want to sleep alone” and “Resists going to bed at night”. Mothers responded to statements about child sleep problems on a 3‐point scale ranging from not true (1), somewhat or sometimes true (2), and very true or often true (3). The standardized factor loadings for the items were 0.77 and 0.62, respectively. The graded response item response theory (IRT) modeling of child sleep problems allows for estimating random measurement error in the outcome. In sibling comparison studies random measurement error is located at the lowest level of analysis, making measurement models particularly beneficial in within family studies.

### Sibling comparison and instrumental variable

**FIGURE 1:**
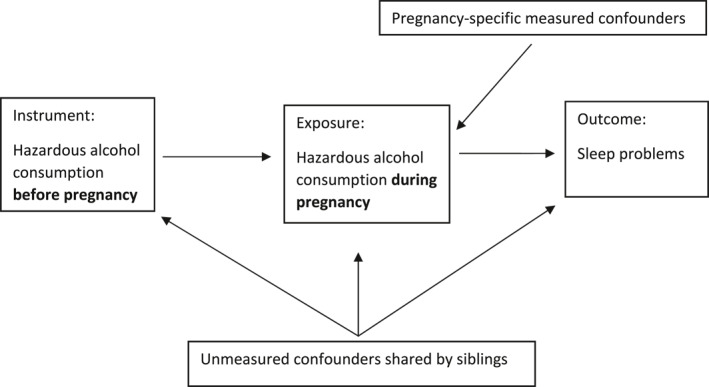
Instrumental variable approach on within sibling pair effects. The instrument is associated with the exposure of interest, but we assume it has no direct effect on the outcome

We used sibling comparison and maternal hazardous drinking during the last 3 months prior to pregnancy as an instrumental variable to adjust for variables that were stable across pregnancies. This approach has been described in previous research (Lund et al., 2019). Sibling comparisons allow comparing outcomes among siblings exposed to different maternal drinking patterns during the first trimester; but who share family background and environment. The instrumental variable approach allows us to determine whether prenatal exposure to maternal hazardous drinking has a causal effect on child sleep problems. Figure 1 illustrates our instrumental variable approach on within sibling pair effects. Instrumental variables are defined by three assumptions: one, the instrument (hazardous drinking before pregnancy) is associated with the exposure of interest (hazardous drinking during pregnancy). Two, no uncontrolled common causes of the exposure and outcome are associated with the instrument. Three, the instrument has no direct effect on the outcome of interest, that is, maternal hazardous drinking is independent of child sleep, given the measured covariates and shared confounders. Within siblings of the same mothers, shared unmeasured familial confounding is controlled by design, so although the instrumental variable assumptions are not formally testable, it is likely that these assumptions hold. The sibling comparison approach represents a within‐person design at the exposure (i.e., maternal) level. Hence, we assume that prenatal alcohol consumption is associated with the outcome on the between‐person level but with no direct effect on the outcome on the within‐person level. By nesting the instrumental variable approach within the sibling comparison design and adjusting for observed confounders, including birth order, we assume that it is possible to evaluate the causal effect of hazardous drinking during pregnancy on child sleep problems.

### Covariates

From the MBRN we included parity, and from MoBa questionnaire we included unplanned pregnancy, daily smoking, and pre‐pregnancy alcohol abstinence as covariates. These potential confounders can vary across pregnancies, and they are not likely consequences of hazardous drinking or alcohol use disorders.

### Statistical analyses

We used a graded response item response theory model (GRM‐IRT) to model the sleep problem scale of the CBCL; this is a confirmatory factor analysis for ordinal items using a logit link function. Hence, each response category of each item has a separate threshold, and each item has a single slope (i.e., factor loading). To ensure interpretability, we used the CBCL standard of the T‐score (SD of 10) and fixed the variance at the first time point to this value. We used a latent growth curve to model sleep problems across time. The growth curve had its random intercept set to 1.5 years and a fixed slope (30). To model changes in latent means and variances across time, we set the thresholds for each response category and factor loadings for each item to equal across time.

In three steps, we regressed the latent intercept and slopes of the growth curves on the hazardous drinking measures and the covariates. First, we adjusted for the covariates. Second, we adjusted for the group mean‐centered hazardous drinking measures to perform sibling control analyses. Third, we exchanged the exposure to hazardous drinking 3 months before pregnancy and adjusted for the group mean‐centered hazardous drinking 3 months before pregnancy as an instrumental variable by regressing hazardous drinking during pregnancy on hazardous drinking before pregnancy in a structural equation model. We used the default Sandwich estimator of Mplus 7.31 to correct for dependent observations in the sibling data. We used Full Information Maximum Likelihood (FIML), thus all available information was used to estimate the model. Using FIML to handle missing data, all cases where at least one outcome time‐point is included under the Missing At Random assumption. Our choice to model both time points in a single model renders the advantage of (FIML) estimation of potential bias from non‐response at either time point correlated with any observed covariate included in the model or unobserved time‐invariant maternal factor correlated with such non‐response.

## RESULTS

Table 1 illustrates the characteristics of the pregnant women who participated in MoBa with two or more pregnancies. The mean age at recruitment was 30 years, and most pregnancies were planned (83.2%). Daily smoking during pregnancy was reported by 6%. While about half (49.7%) reported hazardous drinking during the 3 months prior to pregnancy, only 2.49% did so during the first trimester.

**TABLE 1:** Characteristics of the 30,387 pregnancies of 15,910 mothers from the Norwegian Mother and Child Cohort study

Mean age at gestational week 17/18 in years (SD)	29.99 (4.18)
Parity (%)	
*0*	11,636 (38.3)
*1*	13,356 (44.0)
*2*	4228 (13.9)
*3*	873 (2.9)
*4 or more*	294 (1.0)
Unplanned pregnancy (%)	
*No*	25,013 (83.3)
*Yes*	5374 (17.7)
Daily smoking during pregnancy (%)	
*No*	28,401 (93.5)
*Yes*	1986 (6.5)
Pre‐pregnancy abstinence from alcohol (%)	
*No*	29,343 (96.7)
*Yes*	1044 (3.4)
Hazardous alcohol consumption^a^	
During the 1^st^ trimester (%)^b^	
*No*	28,935 (96.4)
*Yes*	1088 (3.6)
During the 3 months prior to pregnancy (%)	
*No*	12,534 (41.3)
*Yes*	17,853 (58.8)

^a^ Hazardous drinking was defined as scoring 3 or more on AUDIT‐C.

^b^ *n* = 30,023.

Hazardous drinking during the first pregnancy (4.7%) was more common than in following pregnancies (2.4%). Among women that did not report hazardous drinking in the second pregnancy, 4.2% did so in the previous pregnancy. Conversely, among women who did not report hazardous drinking in the first pregnancy, 1.8% did so in the following pregnancy. The relative risk for a woman with hazardous drinking during pregnancy to also report hazardous drinking in the following pregnancy was estimated to 7.41 (95%CI 5.89–9.33). This estimate was not altered after adjusting for parity and maternal age (RR = 7.43; 95%CI 5.90–9.35). The tetrachoric correlation between hazardous drinking over two sequential pregnancies were estimated to *r* = 0.47.

Table 2 shows the results from the regression analysis of child sleep problems for audit scores 1, 2, and ≥ 3. In the unadjusted analyses, hazardous drinking during pregnancy (i.e., an AUDIT score ≥ 3) was associated with sleep problems at 1.5 (*β* = 1.14, 95%CI 0.04–2.25) and 3 (*β* = 2.86, 95%CI 1.85–3.87) years. In analyses adjusted for measured confounders, hazardous drinking during the first trimester was still associated with sleep problems at 3 years (*β* = 2.50, 95%CI 1.46–3.57). We adjusted for maternal risk factors stable across pregnancies by sibling control, which markedly reduced the associations between maternal hazardous drinking and child sleep problems. When pre‐pregnancy drinking was included as an instrumental variable, the effects of hazardous drinking during pregnancy on sleep problems were non‐significant at 1.5 (*β* = −0.32, 95%CI −1.91–1.26) and 3 years (*β* = 0.06, 95%CI −1.56–1.64).

**TABLE 2:** Results from latent growth analyses on the effect of maternal hazardous drinking during pregnancy on child sleep problems at age 1.5 and 3 years

	Unadjusted						Model 1: Adjusted						Model 2: Sibling control						Model 3: Sibling and instrumental variable control					
	1.5 years			3 years			1.5 years			3 years			1.5 years			3 years			1.5 years			3 years		
	β	95% CI		β	95% CI		β	95% CI		β	95% CI		β	95% CI		β	95% CI		β	95% CI		β	95% CI	
AUDIT = 1	0.18	−0.38	0.73	1.67	1.17	2.16	0.16	−0.40	0.73	1.59	1.09	2.10	−0.24	−1.05	0.57	0.42	−0.35	1.18	−0.10	−0.87	0.68	0.61	−0.12	1.35
AUDIT = 2	1.04	0.26	1.81	1.36	0.62	2.11	0.89	0.10	1.68	1.07	0.32	1.83	0.53	−0.65	1.71	−0.20	−1.33	0.93	0.85	−0.28	1.97	−0.16	−1.24	0.93
AUDIT ≥ 3	1.14	0.04	2.25	2.86	1.85	3.87	0.84	−0.30	1.97	2.50	1.46	3.54	−0.70	−2.32	0.93	−0.29	−1.91	1.33	−0.32	−1.91	1.26	0.06	−1.53	1.64

Model 1 adjusted for observed covariates: parity, unplanned pregnancy, daily smoking, and pre‐pregnancy alcohol abstinence. Model 2 adjusted for familial risk factors by a sibling control design. Model 3 adjusted for non‐shared environmental risk factors by using hazardous drinking in the 3 months prior to pregnancy as an instrumental variable.

## DISCUSSION

To disentangle the effect of prenatal exposure to alcohol on sleep outcomes in young children, we compared sleep outcomes in siblings raised in the same family that was differently exposed to alcohol during pregnancy, and we used hazardous drinking before pregnancy as an instrumental variable. Initially, in the unadjusted model, we found that hazardous drinking during pregnancy was associated with offspring sleep outcomes at 1.5 and 3 years. In the adjusted model, there was an association between maternal hazardous drinking and sleep problems when children were 3 years old. However, with sibling control and instrumental variable, the effects were greatly reduced and insignificant. The results did not show a putative causal effect of hazardous drinking during the first trimester on child sleep problems at ages 1.5 and 3 years.

To our knowledge, this is the first study that has used sibling control when examining the effect of prenatal exposure to alcohol on offspring sleep problems. Therefore, our findings cannot be discussed in the context of studies using an equally robust research design. However, comparing our findings with previous studies, utilizing a non‐causal design, findings from our initial adjusted model, are in line with findings from several studies that showed an association between prenatal exposure to alcohol and child sleep problems (Alvik et al., 2011; Chandler‐Mather et al., 2021; Harskamp‐van Ginkel et al., 2020; Pesonen et al., 2009; Shang et al., 2006; Troese et al., 2008). An Australian longitudinal study showed that children of women who drank heavily during pregnancy were at increased risk of sleep problems from ages 2–9 (Chandler‐Mather et al., 2021). A Norwegian population‐based longitudinal study found that maternal binge drinking weekly or more the first 6 weeks of pregnancy was associated with infant sleep problems (Alvik et al., 2011). A Finnish study found that children exposed to more than 12 g of alcohol per week during pregnancy had increased risk of shorter sleep duration and lower sleep efficiency at age 8 (Pesonen et al., 2009). A Dutch cohort study showed that children of mothers who consumed 1 or more units of alcohol per day 14 weeks into pregnancy had increased risk of sleep problems at ages 7–8 (Harskamp‐van Ginkel et al., 2020).

The findings from our more comprehensive models, that utilized sibling control and instrumental variable, were not in line with the abovementioned studies; our final models did not suggest a causal effect between maternal hazardous drinking during the first trimester of pregnancy and child sleep problems at ages 1.5 and 3 years. These findings were more in line with a Finnish cohort study that did not show an association between prenatal alcohol exposure and sleep problems infants (Morales‐Muñoz et al., 2018), and a longitudinal study from the United States, where maternal drinking during pregnancy was not associated with child sleep problems the first 12 years of life (Stone et al., 2010). All maternal risk factors in the Finnish study (e.g., anxiety, depression, insomnia, attention deficit and hyperactivity disorder), except alcohol use during pregnancy, were associated with infant sleep problems (Morales‐Muñoz et al., 2018), and of all the maternal risk factors in the U.S. study, prenatal exposure to opiates, cocaine, marijuana, alcohol, and nicotine – only nicotine was a unique predictor of child sleep problems (Stone et al., 2010). While these studies did not utilize designs that allowed making statements about causal inference, their adjusting for several psychiatric and substance use problems/disorders, achieved much the same as our study in controlling for common genetic factors which may confound the association between maternal drinking during pregnancy and offspring sleep problems.

Maternal alcohol use during pregnancy is associated with several environmental risks, including maternal psychiatric and substance use problems, which are also associated with offspring psychiatric problems (Kendler et al., 2003), including sleep problems (Moore et al., 2011). Further, genetic factors influence both maternal alcohol use during pregnancy and a range of other behaviors (Kendler et al., 2003), including sleep problems (Wang & Saudino, 2012). Thus, genetic confounding may account for the association between maternal hazardous drinking during pregnancy and offspring sleep problems, observed in our unadjusted and adjusted models and several previous studies (Alvik et al., 2011; Chandler‐Mather et al., 2021; Harskamp‐van Ginkel et al., 2020; Pesonen et al., 2009; Shang et al., 2006; Troese et al., 2008). As noted in a recent review, failing to account for genetic factors may lead to biased estimates and erroneous conclusions in studies on associations between parental characteristics and child mental health (Jami et al., 2021). The findings of the present study illustrate this point nicely; initial models suggested that prenatal exposure to maternal hazardous drinking was associated with offspring sleep problems, but when controlling for familial confounding in the final models, a different picture emerged; suggesting that the association is not causal, but due to familial confounding.

## METHODOLOGICAL CONSIDERATIONS

Key strengths of the study include that it is based on a prospective population cohort study with a large sample size. It utilizes several quasi‐experimental approaches to control for unmeasured confounding. Further, we used a validated measure for assessing maternal drinking (Bradley et al., 2003; Bush et al., 1998), women reported pre‐pregnancy and first‐trimester drinking twice, with high consistency regarding how often and how much alcohol they consumed. We used FIML to handle missing data, thus all cases with data on at least one outcome time‐point are included under the Missing At Random assumption. Important limitations that should be considered when interpreting the findings are the MoBa participation rate and that some groups are underrepresented, including women who live alone, are under 25 years old, smoke, and women that have previously given birth to more than two children. However, compared to the general pregnant population in Norway, differences are small (Magnus et al., 2016; Nilsen et al., 2009). It is not optimal that sleep problems are measured using only two questions. Further, we only have maternal reports on offspring sleep problems, which increases the risk of common method bias; it would be preferable with information from other sources too, for example, from wearables, including additional aspects of offspring sleep problems. Further, in our study, differentially exposed children were, by design, balanced on stable factors leading to selection, such as maternal socioeconomic status. It would have been preferable also to have information on child sleep problems from multiple sources. Previous studies could, in different ways, have been confounded by systematic rating bias. Crucially, a major strength of our within‐family sibling control analysis is that any between‐family maternal rating bias is accounted for by design. Sibling designs increase the standard error of the estimates, and we cannot rule out that our study lacked sufficient power to detect small but true effects. The attenuation of associations due to random measurement error in exposure is also higher, and therefore weaker, in within‐pair estimates (Frisell et al., 2012). However, the IRT modelling of random measurement in the outcome is a particular strength of the study, since such error is located at the lowest level of analysis (i.e. specific pregnancy‐child) and would otherwise deflate any exposure‐outcome estimates. Despite the abovementioned limitations, sibling designs are considered a useful approach to exploring possible causality. This is especially true when, as in our study, it is combined with other quasi‐experimental approaches that control unmeasured confounding (Frisell et al., 2012).

## CONCLUSION

Our findings suggest no causal effect of maternal hazardous drinking during the first trimester on child sleep problems at ages 1.5 and 3 years. The findings underscore the importance of utilizing methods accounting for familial confounding in studies on associations between parental exposures and child outcomes and that failing to do so may lead to erroneous conclusions. The findings should not be interpreted as implying that maternal alcohol use during pregnancy is not harmful; It is well established that maternal drinking during pregnancy is associated with other harmful outcomes for children, including fetal alcohol syndrome and fetal alcohol spectrum disorders (Lange et al., 2017; Popova et al., 2018).

## AUTHOR CONTRIBUTIONS

Dr. Ingunn Olea Lund assisted in study conceptualization and study design, assisted in data analysis aspects, interpreted results, drafted the initial manuscript, and reviewed and revised the manuscript. Dr. Eivind Ystrom conceptualized and designed the study, designed the data analysis strategy, conducted the data analysis, assisted in drafting the initial manuscript, and reviewed and revised the manuscript. Both authors approved the final manuscript as submitted and agree to be accountable for all aspects of the work.

## CONFLICTS OF INTEREST

Eivind Ystrøm is a Joint Editor for JCPP Advances. Ingunn Olea Lund has declared no competing or potential conflicts of interest.

## ETHICS STATEMENT

All study participants provided written informed consent. MoBa has a license from the Norwegian Data Inspectorate, and this study was approved by the Regional Committee for Medical Research Ethics.

## Data Availability

The consent given by the participants does not open for data storage on an individual level in repositories or journals. Researchers who want access to data sets for replication should apply datatilgang@fhi.no. Access to data sets requires approval from The Regional Committee for Medical and Health Research Ethics in Norway and an agreement with MoBa.
